# Occupational therapy services in primary care: a scoping review

**DOI:** 10.1017/S1463423622000123

**Published:** 2023-01-09

**Authors:** Catherine Donnelly, Leanne Leclair, Carri Hand, Pamela Wener, Lori Letts

**Affiliations:** 1 School of Rehabilitation Therapy, Queen’s University, Kingston, Ontario, Canada; 2 Department of Occupational Therapy, University of Manitoba, Winnipeg, Manitoba, Canada; 3 School of Occupational Therapy, Western University, London, Ontario, Canada; 4 School of Rehabilitation Science, McMaster University, Hamilton, Ontario, Canada

**Keywords:** primary care, primary health care, occupational therapy, scoping review

## Abstract

**Aim:**

To examine and describe the current evidence about occupational therapy services in primary care.

**Background:**

Interprofessional primary care teams have been introduced to support the changing demographics and provide more comprehensive and coordinated care. Occupational therapists have the opportunity to play an important role in this expanding area of practice. To do so, occupational therapists must develop roles built on evidence and a clear understanding of the care delivery context.

**Methods:**

A scoping review was conducted based on the scientific and grey literature. Studies that described or examined the occupational therapy role with clients (individuals, groups, communities, populations) of all ages, conditions or occupational issues in a primary care context and that presented or referred to an occupational therapist working in a primary care setting were included. Studies were excluded if they were not in English or French. The Canadian Model of Occupational Performance and Engagement was used to chart the data.

**Findings:**

129 articles were identified, with 62 non-research and 67 research-focussed articles. A total of 268 assessments and 868 interventions were identified. The top interventions offered by occupational therapists were referring to/advocating for/coordinating/linking to and navigating community services (*n* = 36 articles), chronic disease management (*n* = 34 articles)/self-management education (*n* = 28 articles), health promotion (*n* = 30 articles) and falls prevention (*n* = 27 articles). The predominant focus in the literature is on adult and older adult populations.

## Introduction

Primary care has undergone significant transformation in the past 20 years. Around the globe, populations are ageing, and health care systems are shifting focus from acute care to chronic disease management (Public Health Agency of Canada, 2017; College of Family Physicians of Canada, 2019). There is a recognition that increasingly complex patients require the support of interprofessional primary care teams for the provision of comprehensive and coordinated care (Hutchison *et al*., 2011; Somé *et al*., 2020). Most notably during the pandemic, primary care teams have demonstrated an important role in supporting individuals to cope with the primary care secondary impacts of COVID-19 (Ashcroft, Donnelly, Dancey, *et al*., 2021; Ashcroft, Donnelly, Gill, *et al*., 2021; Donnelly *et al*, 2021). As greater numbers of professions work in primary care settings, it is critical to articulate roles and identify evidence, or lack thereof, to support and ensure best practices in interprofessional primary care teams (Donnelly *et al*., 2013; Brown *et al*., 2021).

The vast majority of individuals address their health care needs in primary care settings (Jaakkimainen *et al*., 2006; Kringos *et al*, 2015), and as interprofessional primary care teams expand, opportunities for patients to access occupational therapy in primary care increase (Somé *et al*., 2020). People with or at risk of impairment and disability are best supported in primary care, and growing evidence indicates that occupational therapy’s unique lens can support these individuals in a primary care setting (Richardson *et al*., 2012; Garvey *et al*., 2015; Bolt *et al*,. 2019; Brown *et al*, 2021). Occupational therapists are educated as generalists bringing expertise to help individuals of all ages and develop, recover and improve as well as maintain function and skills needed for daily living. They promote engagement in occupations that influence an individual’s health and well-being (Law *et al*., 1998). Occupations refer to the “everyday activities that people do as individuals, in families and with communities to occupy time and bring meaning and purpose to life. Occupations include things people need to, want to and are expected to do.” (World Federation of Occupational Therapists, n.d.).

Occupational therapy associations, including those in Canada, the United States, Australia and Europe, are recognizing the importance of articulating the profession’s role in primary care to support interprofessional primary care best practices (Bolt *et al*., 2019; Canadian Association of Occupational Therapists [CAOT], 2013; Muir, 2012; American Occupational Therapy Association [AOTA], 2020). However, one of the biggest challenges to occupational therapy’s integration in primary care is the lack of understanding of their role by other primary care team members (Donnelly *et al*., 2013; Brown *et al*., 2021). Historically, and most notably, occupational therapy has been considered synonymous with rehabilitation, facilitating recovery from and adaptation to any injury, illness or disease including chronic diseases. However, the scope of occupational therapy goes well beyond rehabilitation to include health promotion, disability and disease prevention and community development (Metzler *et al*., 2012; AOTA, 2020); perspectives that are well aligned with primary care. A recent review paper examined occupational therapy interventions in primary care (Bolt *et al*., 2019); however, a broader review of the literature is needed to fully understand the role occupational therapists can play on primary care teams. The objective of this scoping review is to examine and describe the current evidence about occupational therapy services in primary care.

## Methodology

The authors (CD, LL, CH, PW, LL) conducted a scoping review following Arksey and O’Malley’s (2005) methodological framework with the modifications and enhancements suggested by Levac *et al*. (2010). A scoping review provides an overview of the written evidence that is available on a particular topic and typically does not focus on the methodological quality of the existing evidence (Peters *et al*., 2015). Not assessing for methodological quality of the evidence is appropriate when the topic of interest is emergent, and the desire is to include all of the relevant information. Scoping reviews are particularly useful for bringing together research and non-research information. Arksey and O’Malley’s (2005) six-step process for a scoping review was as follows: 1. developing the purpose and review question(s); 2. identifying relevant studies; 3. selecting studies using an iterative team approach; 4. charting the data incorporating a numerical summary and thematic analysis; 5. collating, summarizing and reporting the results along with the implications for policy, practice or research; and 6. consulting with stakeholders to inform or validate study findings (Arksey & O’Malley, 2005). Arksey and O’Malley (2005) stated that the sixth step is optional and this step was not included. No review protocol exists for this scoping review.

The following research question guided the search: *What is the role of occupational therapy in primary care settings?* A professional health sciences librarian performed a scoping review search of the literature across a number of relevant databases. While searches varied in keeping with the options available within each database, a combination of controlled vocabulary and keyword queries was used as available in each database. The title, abstract and subject heading (if applicable) fields were searched. The subject headings searched were occupational therapy, primary care physicians, family physicians, general practitioners, primary care nursing, community health centres/centres, outpatients, ambulatory care, general practice (exploded) and primary health care (exploded). A series of keyword strategies were created to access literature focussing on the concept of occupational therapy and therapists, family physicians and nurses, ambulatory healthcare and patient care, outpatients, community healthcare and health centers to ensure that all relevant studies were captured in the search (see the detailed OVID Medline search strategies Appendix A). The following databases were searched from their earliest date of coverage through August 31, 2021 Ovid Medline, Ovid EMBASE, SCOPUS, EBSCOhost CINAHL, Cochrane Library and Google Scholar. Guideline databases, NICE and National Guideline Clearinghouse were also searched. General searches on Google Scholar using terms such as “role of occupational therapy in primary care”, “occupational therapy” and “primary care” produced a list of articles that helped to inform the final choice of keywords for the search of the databases. The researchers also included websites of occupational therapy associations and manually searching the reference lists of the selected articles to identify any additional literature.

Studies that described or examined the occupational therapy role with clients (individuals, groups, communities, populations) of all ages, conditions or occupational issues in a primary care context and that presented or referred to an occupational therapist working in a primary care setting were included. Studies were excluded if they were not in English or French. Primary care and the structure of occupational therapy practice in this setting were understood based on the key feature described by Starfield (1994), including care that is the first point of contact, comprehensive, coordinated and provided longitudinally over the lifespan. All existing literature including primary research studies, systematic reviews, scoping reviews, narrative reviews, opinion pieces, letters, guidelines, position papers, reports and service descriptions were included as sources for this scoping review.

**Figure 1.**
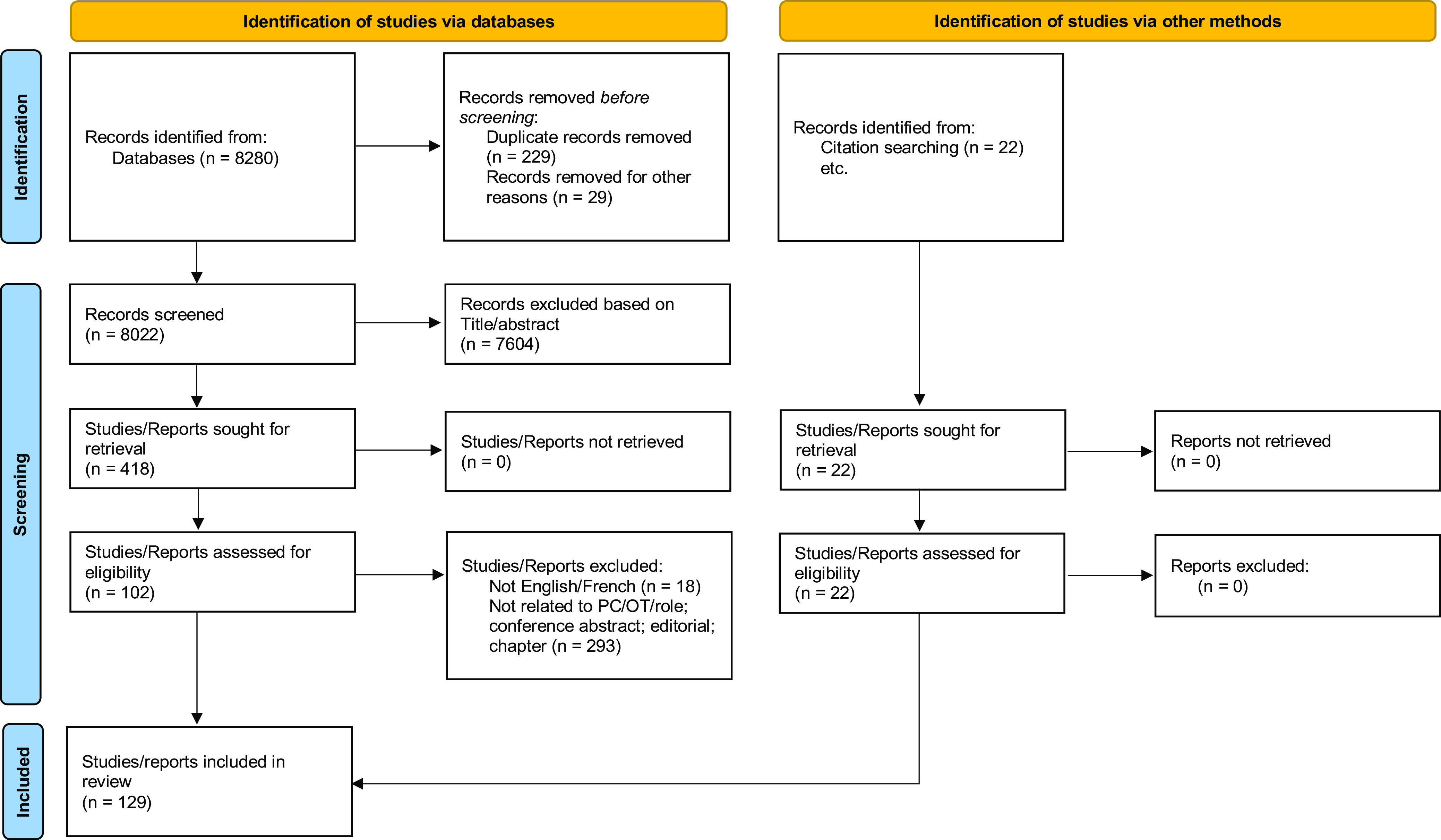
PRISMA 2020 flow diagram for new systematic reviews, which included searches of databases, registers and other sources

There were 8280 documents identified in the initial searches. Documents were divided among the authors of the scoping review and each participated in the independent review of sources based on title and abstract. A team of two authors reviewed every source; in pairs, they compared and discussed the selected sources to ensure agreement. Four hundred and eighteen sources were identified for full-text review through this process. Of these sources, 102 met the selection criteria and were included, 18 were not available in English or French (the languages spoken by the authors) and 293 did not specify the role of occupational therapists and/or were not related to primary care. Twenty-two additional articles were identified by reviewing the reference lists of relevant articles and searching websites of occupational therapy associations for a total of 129 articles. See Figure 1 for the selection algorithm.

Data were extracted using a charting table to ensure extraction consistency among team members (Levac *et al*., 2010). The data charting table (Appendix B) included headings based on Arksey and O’Malley’s (2005) and Peters *et al*. (2015) data charting forms, which were expanded to include extraction of information on the role of occupational therapy in primary care. Health equity was also considered within the data charting table, a pertinent area to assess given the focus on health equity in many primary care settings. If a specific heading did not apply, as was often the case with grey literature, that column was left blank. Data charting was an iterative process in which all the authors of the scoping review (author initials here) participated and discussed throughout the data charting process to ensure that relevant information was being consistently captured for the review.

Levac *et al*. (2010) identified three stages: analyzing the data, reporting the results and applying meaning to the results. Analyzing the data included completing a descriptive numerical analysis and a thematic analysis (Levac *et al*., 2010). The descriptive numerical analysis involved reporting the number of research and descriptive studies, study designs, publication year, study population and study location (Levac *et al*., 2010). A thematic analysis followed qualitative data analysis techniques in which two authors independently coded each article while considering the occupational therapy practice process to reflect whether the approach involved assessment or intervention and if these approaches were unique to the article. In addition, we coded using the Canadian Model of Occupational Performance and Engagement (Townsend & Polatajko, 2013). While this is a Canadian framework, it is widely used internationally to inform occupational therapy practice.

Using the Canadian Model of Occupational Performance and Engagement (Townsend & Polatajko, 2013), occupational therapists may address three domains including, aspects of the person (physical, cognitive, or affective components), environment (physical, social, cultural, institutional), occupation (self-care, productivity, or leisure) or all three, as they seek to optimize a patient’s ability to perform and engage in meaningful occupations. The Canadian Model of Occupational Performance and Engagement was used to classify assessment and intervention approaches that addressed: the person, occupation, environment or a comprehensive person–environment–occupation (PEO) approach. For example, a falls-prevention intervention that addresses balance (aspect of the person), simplifying activities (aspects of occupation) and falls hazards (aspects of the environment) would be classified as a comprehensive PEO approach. Additionally, we applied a lifespan lens and examined assessments and interventions that focussed on four age groups: children and youth; adults; older adults; adults and older adults.

## Results

A total of 129 articles were included in the final sample. Table 1 provides a summary of the research literature. Just over *half* of the articles (*n* = 67) were researched. Of the sixty-seven research articles that were included, 18 applied a mixed or multiple methods design, 12 were qualitative designs, and 37 were quantitative designs. Of the mixed methods studies, seven involved feasibility studies that used a quasi-experimental design (*n* = 7) or randomized controlled trials (RCT) design (*n* = 1) to examine occupational therapy interventions in primary care. The qualitative component of these mixed methods studies primarily used interviews to examine the feasibility of the interventions. Fourteen of the quantitative studies were pilot RCTs or RCTs, nine were quasi-experimental with the remaining 14 being observational studies including retrospective chart review (*n* = 5) and survey studies (*n* = 4).

**Table 1.** Summary of research articles

Authors (Year)	Country	No of participants (Age, Sex – %M/F)	Study design	Study aim/purpose
Bergin and Keegan (2016)	Ireland	1. 30 children were referred for primary care occupational therapy. Male=22 (77.3%) Female=8 (26.7%) Average Age = 6 years old Age Range=7 years old 2. Four primary care occupational therapists (OT) using the Short Occupational Profile (SCOPE) tool with children.	Mixed methods, sequential exploratory design. Quant: Administration of the SCOPE. Qual: Interviews	To explore the use of the SCOPE initial assessment tool in primary care occupational therapy practice with children.
Boakye *et al*. (2016)	Canada	17 representatives of 10 provincial professional colleges and regulatory bodies, with knowledge of working with patients with non-specific lower back pain (LBP).	Qualitative. Document review, interviews	To explore which health care providers could be involved in centralized intake for patients with non-specific low back pain to enhance access, continuity and appropriateness of care.
Brandis and Tuite (2001)	Australia	62 adults were referred by general practitioners (GP) due to risk of falling. No info about age or gender.	Pre-test post-test pilot intervention	To evaluate the effectiveness of the Falls STOP project, a partnership between general practitioners (GPs) and OTs, with the common goal to reduce accidental falls in the elderly. A home visiting service was implemented that included the organization of home modifications, education on fall prevention strategies and referral to other community services.
Brown *et al*. (2021)	Canada	11 residents (54.5% male; ages ranged from 21 to 50 years); 2 years of first-year resident (*n* = 29) referrals (*n* = 129) to therapy services	Mixed methods, convergent parallel design	To explore first-year family medicine residents’ attitudes and behaviours related to working with occupational therapy (OT) and physiotherapy (PT) in a primary care teaching clinic following exposure to these professions within an interprofessional primary care team.
Carlsson *et al*. (2013)	Sweden	33 adults (*n* = 15 control, *n* = 18 intervention) who were off work or working part-time due to psychiatric or musculoskeletal conditions for less than four weeks. 67% women, mean age 46.	RCT	To examine if GPs, with support from an early multidisciplinary assessment carried out in a primary care setting, could help patients to achieve faster and more appropriate rehabilitation to lower the risk of long-term sick leave.
Cassidy *et al*. (2017)	USA	13 community-dwelling older adults aged 66–88 yrs old.	Mixed methods feasibility study. Quant: Pre-test post-test Qual: Interviews.	To examine the feasibility of Aging Well by Design, a Lifestyle Redesign®–inspired intervention for community-dwelling older adults. The original Lifestyle Redesign programme was shortened to 3 months and implemented as a community outreach programme in a primary care setting.
Clemson *et al*. (2014)	Australia	Providers with experience delivering a home safety fall prevention intervention for older adults at risk for falls living in community. Eight OTs, two programme coordinators.	Qualitative. Interviews.	To explore issues underlying the implementation of an occupational therapist-led evidence-based home safety fall prevention intervention within primary care services in Melbourne, Australia.
Connolly *et al*. (2018)	Ireland	12 adults (majority women) living independently in the community, not attending psychiatric services and reporting experiencing stress. Average age was 52.6 years, majority were women.	Mixed method pilot study. Convergent parallel design. Quant : pre-test post-test, Qual : focus groups	To explore the acceptability of a primary care 6-week stREss maNagemEnt and Well-being (RENEW) programme delivered by OTs.
Cook *et al*. (2004)	England	Providers working with in a new service for clients with severe mental illness, with an expanded team including OT. 8 staff – GP, OT, OT support worker, practical nurse, consultant psychiatrist, community psychiatric nurse, residential home manager, residential care worker.	Qualitative component of a mixed methods case study. Interviews.	To investigate the impact of primary care team-based mental health services on key staff and the effects on working relationships and patient care.
Cook (2003)	UK	25 people with enduring psychotic conditions. 88% male, 88% over age 40 years.	Descriptive retrospective chart review	To investigate the type and frequency of occupational therapy and care management interventions delivered to people with severe mental health problems in primary care.
Cook and Howe (2003)	England	32 adults (81% men, 84% age 18-64) with long-term mental illness and impaired social behaviour as a consequence of mental illness (schizophrenia, schizotypal, delusion disorders, affective disorders with psychotic symptoms).	Pre-test post-test feasibility study	To investigate a new model of primary care service that included occupational therapy and was designed to engage clients with enduring psychotic conditions who were not in contact with a secondary care-based community mental health team in an inner-city area.
Cunningham and Valasek (2019)	USA	Three OTs and three women with urinary dysfunction	Case series	To provide preliminary evidence for OT-led interventions targeting urinary dysfunction in coordination with primary care providers.
De Oliveira and Ferigato (2019)	Brazil	Four out of six OTs (the total number of professionals in the Primary Health Care network of a medium-sized city in the interior of the state of Sao Paulo), ages ranged from 30 to 54 years	Qualitative participatory research	To build OT care technologies for the care of women victims of violence and explore how these technologies may come to cope with situations of domestic and family violence in the context of Primary Care from the perspective of OT working in this context
Donnelly *et al*. (2017)	Canada	Three interprofessional primary care clinics. 161 initial COPMs were administered, and 22 were readministered. Average age of participants was 56.7 years (standard deviation [SD = 17], range=23–91; 122 women and 39 men.	Mixed-methods, sequential design Quant: outcome measurement Qual: Interviews	To examine what client issues are being identified through the administration of the COPM (Canadian Occupational Performance Measure) and what is the feasibility of using the COPM as an outcome measure in primary care.
Donnelly *et al*. (2016)	Canada	52 primary care OTs	Cross-sectional survey.	To describe occupational therapy roles and models of practice used in primary care.
Donnelly *et al*. (2014)	Canada	OT and team members at 4 practices (family health teams) including executive director, OT, NP, SW, GP, dietician and diabetes educator.	Qualitative multiple case study. Interviews; document analysis; questionnaires.	To describe the emerging role of occupational therapy in Family Health Teams, a model of interprofessional primary care.
Donnelly *et al*. (2013)	Canada	Four practices (family health teams) with OTs as part of the team.	Qualitative multiple case study Interviews; document analysis;ocusednaires.	To examine how occupational therapy services are being integrated into primary care teams and understand the structures supporting the integration.
Drummond *et al*. (2020)	UK	136/158 individuals met eligibility criteria (ages 31–50 years, 2/3 were women, 2/3 had mental health problems; 30/52 patients returned the questionnaire. 14 patients and 12 stakeholders (GPs *n* = 2, nurse *n* = 1, front-desk staff and practice managers *n* = 2, OTVoc oTs *n* = 4, patient employers *n* = 3) were interviewed.	Mixed methods study	To test the feasibility of delivering OTVoc to provide return to work advice and support for people with musculoskeletal conditions and mental health problems, in primary care
Dunbar and Reed (1999)	USA	Children at risk of developmental or behaviorual problems due to poverty (*N* = 77).	Descriptive. Needs assessment and programme development	To describe services included in primary care to better meet the needs of low-income families with infants and toddlers. Services addressed: 1) developmental milestone screening and intervention, 2) support for child rearing, 3) opportunities for typical play experiences and 4) awareness of disciplinary alternatives.
Eklund and Erlandsson (2014)	Sweden	Women who were employed had a stress-related disorder and had been on sick leave for the past two months or more. Intervention group = 42, care as usual group = 42. Mean age=45.	Pre-test post-test study with matched controls.	To (i) assess the outcomes of the 16-week Redesigning Daily Occupations (ReDO) programme for women on sick leave due to stress-related disorders, in terms of occupational value, satisfaction with everyday occupations, and participation level and (ii) investigate the relationships between those outcomes and return-to-work rate.
Fox *et al*. (2021)	Ireland	ReDO-10 participants *n* = 10 (female, between ages 18-66, diagnosed with anxiety or had stress-related concerns with/without another diagnosis), GP *n* = 9, OTs *n* = 2	Mixed methods feasibility study, multi-phase explanatory sequential design Quant:pre-test post-test intervention Qual:Interviews	To explore the feasibility of a future RCT of the ReDo-10 programme in Ireland and the contextual factors that would influence future implementation
Fritz *et al*. (2020)	USA	60 prefrail older (ages 55+) African Americans. Age=76.6 years, 21 (35%) males, 39 (65%) females. *n* = 29 received control intervention, *n* = 30 received allocated frailty prevention programme intervention, *n* = 1 refused control assignment. For the 30 frailty prevention programme intervention, Age=75.8 years, 10 (33%) males, 20 (66%) females. Study retained 75% (*n* = 45) of participants through the four-month follow-up and analysis.	Feasibility RCT	To evaluate the feasibility and value of a low-dose frailty prevention intervention integrated with primary care among 60 community-dwelling, prefrail older African Americans aged 55+ recruited from a primary care clinic
Gallagher *et al*. (2016)	Ireland	1. OTs working in primary care (survey) *n* = 39. 2. OTs working in community mental health (interviews) *n* = 10.	Multiple methods 1. Survey 2. Interviews	To examine the perceptions of OTs in Ireland with regard to the role of occupational therapy in managing depression in a primary care setting.
Garvey *et al*. (2015)	Ireland	Adults with multi-morbidities (two or more chronic conditions and a minimum of four repeat medications). Intervention group = 26, wait-list control = 24. Median age: I = 65, C = 67.5. Proportion women: I = 65%, C = 63%.	Pragmatic feasibility RCT	To investigate the effectiveness of an occupational therapy-led self-management support programme, OPTIMAL, designed to address the challenges of living with multiple chronic conditions or multimorbidity in a primary care setting.
Gonzalez *et al*. (2015)	Spain	20 women with fibromyalgia. Mean age 40 years.	Pre-test post-test intervention	To evaluate the improvement of activities of daily living (ADL) and quality of life among individuals with fibromyalgia following seven occupational therapy intervention sessions in primary care.
Gudkovs (2011)	Australia	15 adults with depression or anxiety and one other chronic disease. 5 (33%) men 10 (66%) women. Mean age of 54 years.	Pre-test post-test intervention	To evaluate a comprehensive lifestyle programme for “hard to treat ” patients with physical and mental health problems in a rural community primary care team.
Gustavsson *et al*. (2018)	Sweden	65 adults (54 (83%) women and 11 (17%) men, aged 23–63 years) with chronic pain seeking care at six primary health care centres for musculosketal pain where the pain has been persistent for more than three months. 34 (31 (91%) women; 3 (9%) men) intervention + standard care and 31 (23 (74%) women; 8 (26%) men) standard care only. Complete data were obtained for 32 patients (15–- 13 women (87%); 2 (13%) men; intervention + standard care and 17 (14 (82%) women; 3 (18%) men) standard care only).	Pragmatic feasibility RCT	To explore the activity and life-role targeting rehabilitation programme (ALAR) that promotes patient’s active involvement in pain rehabilitation in a primary care setting.
Hand *et al*. (2011)	Canada	1. 16 key stakeholders within occupational therapy for chronic disease management including researchers, OTs, chronic disease organizations, health professional associations and funders. 2. 218 Canadian Association of Occupational Therapists (CAOT) members (survey).	Multiple methods 1. Scoping review and internet scan. 2. Consensus meeting–- Exchange Event 3. Survey of OTs	To develop an agenda of priority areas within collaborative chronic disease research to which occupational therapy can make a contribution.
Hansson *et al*. (2009)	Sweden	13 adults with stress-related disease. 10 (77%) women, 3 (23%) men, median age 40 years.	Retrospective cohort.	To describe a multidisciplinary programme, given by an OT and a PT, for patients with stress-related disease in primary care and to measure the effect of this programme in terms of self-perceived health, degree of burnout, physical activity, symptoms, recreational activities, and psychological and physical well-being.
Holmqvist *et al*. (2014)	Sweden	405 OTs from 4 types of practice settings, one of which was primary care.	Survey	To describe Swedish OT practice patterns for clients with cognitive impairment following acquired brain injury.
Johansson *et al*. (2018)	Sweden	Community-dwelling older adults over 65 years with one or more accidential falls in the last six months, one or more fall-related incident (trip, slip, stumble) or concerns about falling. Intervention group *n* = 74, mean age 77 years, 20% male; Control group *n* = 57, mean age 76 years, 11% male.	Pilot RCT	To compare and evaluate a multifactorial falls-prevention programme, with ordinary falls prevention in primary care.
Johansson and Björklund (2016)	Sweden	Older adults (60+ years) with disabilities participating in in-home rehabilitation in primary care. Quasi-experimental: control = 11, 9 (82%) women; 2 (18%) men; intervention = 8, 6 (75%) women; 2 (25%) men. Interviews: 3 individuals from intervention group.	Mixed methods pilot study. Quant: pre-test post-test design, non-equivalent control group. Qual: Interviews.	To investigate whether a four-month occupation-based health-promoting programme for older persons living in community could maintain/improve their general health and well-being.
Johansson and Björklund (2006)	Sweden	Community-dwelling older adults. Intervention = 22, 21 (95%) women, mean age 82 years. Control = 18, 17 (94%) women, mean age 81 years.	Mixed methods. Quant: pre-test post-test, Qual: Interviews, content analysis based on Model of Occupational Adaptation.	To examine whether the use of the Occupational Adaptation Model increased independence and improved health of older adults with disabilities participating in primary care home rehabilitation.
King *et al*. (2006)	United States	Adults with type 2 diabetes. Intervention *n* = 153, control *n* = 148. Average age 61.5 years, 50% female.	Randomized controlled trial (RCT)	To evaluate the effectiveness of a multifaceted physical activity intervention for people with type 2 diabetes that emphasized participant choice in activity selection. Baseline activity patterns were examined to determine whether they predicted changes in physical activity at two months.
Lamarche *et al*. (2020)	Canada	21 Primary care providers, including 3 registered dietitians, 6 OTs, 4 PTs, 1 registered massage therapist, 1 kinesiologist, 4 family physicians, 1 nurse and 1 social worker.	Qualitative interpretive	To explore how primary care providers define body image, including how they see the concept of body image as it relates to their patients and how is the care for body concerns supported in primary care.
Lamb *et al*. (2010)	United Kingdom	Populat705 adults with sub-acute low back pain for at least 6 weeks. Average age 54 years, 60% female. Intervention *n* = 466, control *n* = 233	Multi-site RCT	To estimate the clinical effectiveness of active management (AM) in general practice versus AM plus a group-based, professionally led cognitive behavioural approach (CBA) for subacute and chronic low back pain (LBP) and to measure the cost of each strategy over a period of 12 months and estimate cost-effectiveness.
Lambert *et al*. (2007)	England	Adults with panic disorder, with or without agoraphobia. Intervention *n* = 57 (68% women, mean age 40 years), usual care *n* = 60 (68% women, mean age 39 years).	Pragmatic RCT	To test the hypothesis that modifying habitual lifestyle behaviours in a health-protective direction will reduce the frequency and severity of anxiety and panic symptoms in panic disorder patients compared with usual GP care and report cost-effectiveness.
Locas *et al*. (2019)	Canada	6 primary care physicians (5 women, 1 man)	Qualitative descriptive	To describe the perspectives of physicians who are working in FMGs in Quebec and have a sufficient understanding of OT to articulate what could be the role of OTs within family medicine teams.
Mackenzie *et al*. (2018)	Australia	40 OTs working in primary care. Settings were public health service (31%), hospital (31%) or private practice (27.5%)–- remainder worked in residential care facilities or non-governmental agencies.	Pre-test post-test.	To examine current practice in preventing falls among older people living in the community prior to attending a home safety workshop; explore the outcomes of the workshop on fall prevention practice and investigate self-reported changes in practice three months after the workshop.
Mårtensson and Marklund (1999)	Sweden	70 adults with chronic pain for no more than six months. 87% women, mean age of 48 years.	Pre-test post-test intervention	To evaluate a biopsychosocial rehabilitation programme in primary healthcare for chronic pain patients
McGrath and O’Callaghan (2014)	Ireland	OTs working with people with dementia or carers of people with dementia. 19 worked in primary and community care, 13 in hospital, 4 in rehabilitation, 1 private practice, 7 in nursing home and 3 mixed practice.	Cross-sectional survey	To explore OT assessment and intervention practices for people with dementia in Ireland.
Merryman and Synovec (2020)	USA	12 HCPs, with 75% (*n* = 8) Master’s prepared social workers, (*n* = 1) certified nurse practitioner (CNRP), (*n* = 1) nurse (RN), (*n* = 2) physicians.	Qualitative	Explore provider referrer perceptions of a new OT service for homeless adults in a Federally Qualified Health Centers (FQHC) to assist effective allocation of scarce resources
Middlebrook and Mackenzie (2012)	Australia	Providers working with community-dwelling older adults at risk for falls. 5 private oTs, 5 private PTs.	Qualitative, grounded theory	To investigate the processes involved for OTs and PTs to implement Medicare items from the Enhanced Primary Care programme within their practice for the purpose of fall prevention interventions for older people.
Mirza *et al*. (2020)	USA	18 adult volunteers (*n* = 8 in intervention group, *n* = 10 individuals in control group), ages ≥50 years, with heart disease, arthritis, and uncontrolled diabetes. 7 men and 11 women aged 50–78 years (M = 61.8, standard deviation = 7.3). 10 PCPs completed feedback surveys.	Feasibility RCT	To assess the feasibility of delivering a primary care occupation-focussed intervention (Integrated pRimary care and OT for Aging and Chronic disease Treatment to preserve
Naidoo *et al*. (2017)	South Africa	Community members with disabilities and their families and community health workers. 37 community members (33 (89%) women and 4 (11%) men ranging in age from 19 to 70 years), 23 community health workers (21 (91%) women and 2 (9%) men ranging in age from 31 to 60 years).	Qualitative descriptive	To understand the challenges of being disabled and the services required by OTs in rural communities to better inform the OT training curriculum.
Norberg *et al*. (2017)	Sweden	Two OTs working in primary health care, five community-dwelling clients with chronic heart failure ranging in age from mid-50s to mid 9’'s.	Mixed methods, case study	To describe clients and OTs experiences of a home-based programme focussed on energy conservation strategies for clients with chronic heart failure.
Olsson *et al*. (2020)	Sweden	Non-probability sample of 86 women (response rate 70%) from 5 primary care centres	Longitudinal single cohort	To investigate if the occupation-based intervention ReDOTM-10 predicts workability for women at risk for or on sick leave.
O’Toole *et al*. (2021)	Ireland	8 PC teams; 149 patients intervention *n* = 78–- 25 (32.1%) male; 53 (67.9%) female control *n* = 71–- 21 (29.6%) male and 50 (70.4%) female with multimorbidity, from November 2015 to December 2018. age–- intervention mean = 65.5 (SD 9.3) years ; control 65.9 (SD 10.5) years	Pragmatic RCT	To evaluate the effectiveness of a group-based, 6-week, OT-led self-management support programme (OPTIMAL) in improving QoL for patients with multimorbidity
O’Toole *et al*. (2013)	Ireland	16 participants with multiple chronic conditions. Average age 67 years, 75% female.	Mixed methods pilot study. Convergent parallel design. Quant: Pre-test post-test. Qual: Focus groups	To assess the feasibility and potential impact of an occupation-based self-management programme for community living individuals with multiple chronic conditions.
Pyatak *et al*. (2019)	USA	aged 18–75 years *N* = 142 participants randomized. Intervention *n* = 71: *n* = 51 received intervention with *n* = 39 who completed follow-up, Control *n* = 69; all completed follow-up. 20 staff members completed survey at baseline, and 26 staff members completed it at follow-up. 11 staff members completed Phase 1 interviews. Eight staff members participated in the focus group. Four providers, eight patients, and the OTs providing LR–OT completed case-based interviews.	RCT	To report on the implementation and preliminary clinical outcomes of a Lifestyle Redesign® (LR)–occupational therapy (LR–OT) diabetes management intervention in a primary care clinic
Restall *et al*. (2005)	Canada	Population/Sample: OTs, Physical therapists, Administrators of community and regional health and social service programmes.	Multiple methods. 1. Literature review 2. Focus groups 3. Key informant interviews	The purpose of the study was to identify, through a review of relevant literature, effective OT and physiotherapy interventions for adults delivered in a primary health care context; to develop a conceptual framework and service delivery model for adult OT and physiotherapy in keeping with the philosophy, principles and vision for primary health care in Winnipeg and to propose a pilot project for OT and physiotherapy services in one community access model.
Richardson *et al*. (2012)	Canada	Adults age ≥44 years with at least three physician visits in the past year and one or more chronic conditions (rheumatoid arthritis, back pain, cardiac arrest, heart failure, chronic obstructive pulmonary disease, stroke, diabetes, emphysema, hypertension, multiple sclerosis, osteoarthritis, osteoporosis, Parkinson’s disease, and cerebral vascular accident). Intervention group *n* = 65, control group *n* = 59. Mean age 63 years, 71% women.	Pre-test post-test with control group	To determine whether patients who receive a multi-component rehabilitation intervention, including online monitoring of function with feedback and self-management workshops, showed less functional decline than case-matched controls who did not receive this intervention and to determine whether capacity building initiatives within the Family Health Team promote a collaborative approach to Chronic Disease Management.
Richardson *et al*. (2010)	Canada	Adults ≥44 years with one or more chronic diseases and at risk of functional decline. Intervention group *n* = 152 (98% women, median age category 55-64 years), usual care control group *n* = 151 (94% women, median age category 55–64 years).	RCT	To determine whether adults with a chronic illness within a primary care setting who received a rehabilitation intervention in this setting showed greater improvement in health status and had fewer hospital admissions and emergency room visits compared with adults who do not receive the intervention.
Rovner *et al*. (2020)	USA	*N* = 101; OT intervention *n* = 50; Diabetes Education *n* = 50 Female *n* = 63 (62%); OT intervention *n* = 31 (62%); diabetes education *n* = 32 (63%) Age sample mean = 68.4 (SD 6.4) years; OT intervention mean = 68.2 (6.1) years; Diabetes sEducation Mean = 68.7 (6.7) years	RCT	To compare the efficacy of an OT behavioural intervention vs community health worker (CHW)–delivered diabetes self-management education to improve glycemic control in African Americans with MCI, low medication adherence, and poor glycemic control.
Schepens Niemic *et al*. (2021)	USA	Latino and Hispanic safety-net primary care patients, ages 50 to 64 years. Participants (*N* = 27) demonstrated clinically significant pre-treatment to long-term follow-up.	Uncontrolled pilot trial	To evaluate patients’ long-term health-related outcomes after lifestyle intervention co-led by OT practitioners and Latino community health workers
Schepens Niemiec *et al*. (2019)	USA	1. 40 Spanish-speaking late-midlife rural-dwelling Latinos, receiving care from a rural health clinic in the Antelope Valley of California. Age ranged from 50-64 years, gender not reported. 2. Key stakeholders involved in the intervention.	Mixed methods, feasibility Quant: pre-test post-test Qual: Interviews	To describe a culturally tailored, activity-focussed lifestyle programme, *¡Vivir Mi* *Vida! (¡VMV!),* study design and protocol, as well as implementation challenges. The goal of *¡VMV!* is to improve the health and wellness of at-risk late-midlife rural-dwelling Latino adults within a primary care setting that serves hard-to-reach, safety-net populations.
Schepens Niemiec *et al*. (2018)	USA	37 Spanish-speaking late-midlife rural-dwelling latinos, receiving care from a rural health clinic in the Antelope Valeey of California. Age ranged from 50 to 64 years, 91% female.	Mixed methods feasibility Quant: pre-test post- Qual: Interviews	To determine the feasibility and efficacy of a culturally tailored lifestyle intervention, ¡Vivir Mi Vida! (Live My Life!), an intervention designed to improve the health and well-being of high risk late middle-aged Latino adults and to be implemented in a rural primary care system.
Stoffer-Marx *et al*. 2018	Austria	*N* = 151 participating patients (74 in the combined-intervention and 77 in the routine-care-plus-placebo group) The follow-up assessment was completed by 59 participants (77%) in the intervention group and 69 participants (89%) in the routine-care-plus-placebo group	RCT	To evaluate and compare the effect of a combined, interdisciplinary intervention feasible in both primary and specialist care, compared to routine care plus placebo in patients with hand OA (osteoarthritis)
Sturesson *et al*. (2020)	Sweden	122 patients (72% women, 28% men). 57 (47%) agreed to an OT assessment. 338 patients from other healthcare centres (HCCs) received sickness certificates but only 266 were usable. 40 patients in intervention HCCs received questionnaires and 25 responses were received back. In contrast, 345 patients in other HCCs received the questionnaires with 142 answers.	Pre-test post-test intervention with comparison non-intervention	To evaluate the feasibility of OTs performing supplementary work ability assessments for persons on sick leave to enhance sickness certifcation quality
Synovec (2020)	USA	*N* = 172 homeless or unstably housed	Descriptive, retrospective chart review	To examine the cognitive status and functional performance of a homeless population receiving medical and mental health services at a health care agency specifically for this population to inform practice
Synovec *et al*. (2020)	US	45 participants out of an initial 83 were included in the analysis. They received OT services at the agency and engaged in OT services for at least 60 days (22% females, 78% males, ages 28–65 years)	Descriptive. retrospectivechart review	To describe the initial outcomes of OT services in a Federally Qualified Health Center providing individuals who are homeless or transitioning into housing access to functional-based interventions to increase self-management and home and community participation.
Tinnelly and Byrne (2016)	Ireland	115 primary care OTs.	Cross-sectional survey	To examine OTs current practices and perceptions of practice, including perceived facilitators and barriers to practice in primary care settings.
Tracy *et al*. (2013)	Canada	42 older adults age 65 years or older with multi-morbidities. 71% of women, mean age 84 years.	Descriptive, retrospective chart review	To design and evaluate a new interprofessional model of care clinic (Interprofessional Model of Practice for Aging and Complex Treatments – IMPACT) for community-dwelling older adults with complex health care needs. A secondary objective was to explore the potential of this new model as an interprofessional training opportunity.
Tran *et al*. (2020)	Canada	*N* = 40	Mixed Methods Feasibility RCT Qual: Interviews	To explore the feasibility of conducting a RCT of an OT-led mindfulness-based stress reduction programme within primary care.
Trembath and Dahl-Popolizio (2019)	USA	Primary care (PC) practice used for the study had 23 practitioners located in four offices across the Phoenix, AZ, metropolitan area.	Descriptive, retrospective chart review	To identify the 15 most common diagnoses in a specific primary care practice and determine how many of them have evidence-based OT interventions appropriate for their treatments
Usher *et al*. (2021)	Ireland	26 HCPs (16 OTs and 10 PTs) completed the pre-Extension for Community Healthcare Outcomes (ECHO) questionnaires, with 96.15% (n = 25) of the respondents being female. Sixteen participants completed the focus group interviews. 21 participants completed the post-ECHO evaluations.	Mixed methods Quant: Survey Qual: Interviews	To enhance palliative care provision in Ireland by facilitating palliative care specialists to disseminate core principles and best practices to primary care HCPs, thus developing communities of practice through Project ECHO.
White *et al*. (2020)		1045 patients receiving care at a free primary care clinic	Multiple methods, 1. retrospective chart review 2. staff and client survey	To examine the pattern of OT referrals and services.

The majority of the publications were from five countries including the United States (*n* = 16), Canada (*n* = 14), Sweden (*n* = 12), Ireland (*n* = 10), England/UK (*n* = 6) and Australia (*n* = 5) with 42% articles published in the past five years and 78% in the past 10 years. Of note, 57% (*n* = 8) of the RCTs were published in the last five years. Included in the non-research publications are six occupational therapy professional bodies that have published descriptions of the role of occupational therapy in primary care (American Occupational Therapy Association [AOTA], 2014; AOTA, 2020; Baaiken *et al*., 2016; CAOT, 2013; Leclair *et al*., 2005; Ontario Society of Occupational Therapists, n.d.; Society of Alberta Occupational Therapists, 2013). The research studies focussed heavily on individuals with chronic conditions, including both physical health and mental health conditions such as pain, diabetes, depression and anxiety. The focus of these studies was primarily on adults and older adults, with only two studies involving children or youth.

Across the research and non-research literature, 268 assessments were identified, of which 139 were unique assessments (i.e. mentioned in only one article) and 868 total interventions were identified, representing 324 unique interventions (i.e. mentioned in only one article) implemented in primary care settings. In the following sections, the assessment and intervention categories are further broken down to examine whether occupational therapy services provided directly in primary care settings focussed on aspects of the person, occupation, environment or all three comprehensively. Supplementary files display the role descriptions according to these categorizations based on non-research articles (position statements, practice guidelines, commentaries and programme descriptions) and research articles. Analysis of this literature shows that overall, occupational therapy in primary care works towards optimizing patient participation in daily occupations including care for self, employment, leisure and social and community activities.

### Assessment

Across all articles, occupational therapy assessment in primary care focussed on the person (36%) with the most frequency, followed by occupation (32%), the environment (26%) and a combination of all three (6%). For those assessments focussed on aspects of the person, developmental screening was the primary assessment identified for children and youth (*n* = 12 unique articles). Person-focussed assessments for adults predominantly addressed physical health domains, including range of motion, strength and endurance, with assessments of mental health depression and anxiety described in six articles. For older adults, person-focussed assessments primarily targeted similar physical health domains as for adults, as well as cognitive screening (*n* = 13 articles).

Assessments of occupation included two specifically for children, one of which involved the observation of play (Dunbar & Reed, 1999). For adults and older adults, the majority of the occupation-focussed assessments examined the analysis of everyday activities (*n* = 20 articles) and self-care (*n* = 19 articles), including both activities of daily living (*n* = 8 articles) and instrumental activities of daily living (*n* = 4 articles).

At the level of the environment, the majority of assessments in primary care targeted the general physical environment (*n* = 38 articles), with home safety assessment (*n* = 19 articles), ergonomic assessments (*n* = 7 articles) and home accessibility (*n* = 6 articles) being the most frequently identified assessments for adult and older adult populations. Falls screening/falls risk assessment (*n* = 9 articles) addressed all of the person–occupation–environment domains. Details of the assessments conducted in primary care can be found in Tables 2 and 3 and supplementary files.

**Table 2.** Summary of assessments and interventions

	Person Research	Person Non	Total Person	Occupation Research	Occupation Non	Total Occupation	Environment Research	Environment Non	Total Environment	PEO Research	PEO Non	PEO Total	TOTAL
Assessment	60	36	96 (36%)	63	28	87 (32%)	30	41	71 (26%)	5	5	15 (6%)	268
Intervention	81	89	170 (19%)	114	137	251 (29%)	89	152	241 (28%)	63	143	206 (24%)	868
TOTAL			266			338			312			221	

PEO = Person–Environment–Occupation.

**Table 3.** Summary of assessments by lifespan

	Person Research	Person Non	Total Person	Occupation Research	Occupation Non	Total Occupation	Environment Research	Environment Non	Total Environment	PEO Research	PEO Non	PEO Total
Children and Youth	6	9	15	3	0	3	2	3	5	0	0	0
Adults	17	0	17	14	0	14	5	9	14	0	0	0
Older Adults	15	0	15	12	7	19	14	1	15	5	4	9
Adults/Older Adults	21	17	38	34	11	45	9	25	34	0	1	1
Lifespan	1	10	11	0	6	6	0	3	3	0	5	5

### Intervention

Occupational therapy interventions addressing occupations were identified with the most frequency (29%), including interventions supporting individuals to engage in self-care, work and leisure activities. Intervention focussing on the environment was the next most frequently reported intervention (28%), including facilitating home safety and accessibility, followed by comprehensive interventions that addressed the person–occupation–environment (24%). Interventions that targeted the person (e.g. exercise) were described with the least frequency (19%). Only 2% of all interventions targeted children and youth.

The majority of interventions in primary care to address aspects of the person for adults and older adults involved exercise, physical activity and movement (*n* = 34 unique articles). Interventions to support mental health were the next most frequently identified interventions and included counselling and psychotherapy (*n* = 10 articles) and anxiety management (*n* = 5 articles). Interventions focussing on cognition identified the use of cognitive behavioural techniques (*n* = 9 articles) and interventions that addressed all components of the person included promoting positive health behaviours (*n* = 8 articles). Only one person-level intervention was identified specifically for children and youth; this intervention was aimed at facilitating typical movement patterns.

Occupation-focussed interventions in primary care for adults most frequently targeted the worker role (*n* = 20 articles), seeking to support individuals to enter, remain or return to the workplace. For adults and older adults, interventions that sought to support functional and community mobility, including driving (*n* = 23 articles) were identified most frequently, followed by lifestyle interventions to modify lifestyles (*n* = 18 articles) and facilitate healthy habit, roles and routines. Occupation-based goal-oriented interventions (*n* = 19 articles) were used across the lifespan. Fewer occupation-level interventions focussed only on older adults, and those that did address supporting healthy eating and nutrition (*n* = 3 articles) and engaging in social activities (*n* = 2 articles).

At the environment, four unique interventions addressed children and youth including school accessibility (*n* = 1 articles), advocacy for inclusive education (*n* = 1 article), facilitating school board requirements and resources (*n* = 3 articles) and supporting parent–child interactions (*n* = 1 article). Interventions that focussed on the environment for adults included workplace ergonomics (*n* = 6 articles) and modifications (*n* = 2 articles). Facilitating home safety was the most frequent intervention for older adults (*n* = 15 articles). The majority of environment-focussed interventions (46%) were applied across all age groups, with the most frequent including referring to/advocating for/coordinating/linking to and navigating community services (*n* = 36 articles), followed by supporting family and caregivers (*n* = 25 articles), and providing assistive technology and adaptive equipment (*n* = 19 articles).

The three most frequently identified interventions for adults and older adults targeted a combination of the PEO, including chronic disease management (*n* = 34 articles), self-management education (*n* = 28 articles) and pain management (*n* = 22 articles). Chronic disease management was the umbrella term used to capture occupational therapy interventions for both physical and mental health conditions and self-management was a more specific description used on its own or along with the description of chronic disease management. Falls prevention was the most frequently identified intervention for older adults and also focussed on the person, occupation and environment together (*n* = 27 articles). A number of other interventions were provided across the lifespan and included health promotion (*n* = 30 articles) and disability and disease prevention (*n* = 17 articles). It should be noted that health promotion and disability and disease prevention were general terms used in the literature and did not include specific details. Full details of the interventions can be found in Tables 2, 3 and 4 and supplementary files.

**Table 4.** Summary of interventions by lifespan

	Person Research	Person Non	Total Person	Occupation Research	Occupation Non	Total Occupation	Environment Research	Environment Non	Total Environment	PEO Research	PEO Non	PEO Total
Children and Youth	2	2	4	2	7	9	3	3	6	0	0	0
Adult	16	3	19	33	17	50	22	8	30	2	0	2
Older adult	17	0	17	18	1	19	17	28	45	7	26	33
Adults/older Adults	45	57	102	53	86	139	32	16	48	49	63	112
Lifespan	1	27	28	8	26	34	15	97	112	5	54	59

### Health equity and access

Access and equity are important issues to consider if our vision is to ensure patients and communities have access to occupational therapy in primary care settings. Two articles (Murphy *et al*., 2017; Marval, 2018) focussed explicitly on health equity issues. Marval (2018) described specific strategies to reduce access barriers for persons who are street-involved, at risk of homelessness or experiencing homelessness in a Canadian context. Murphy and colleagues (2017) explored how occupational therapists in the United States are well-positioned to work with Federally Qualified Health Centres to support access to medically underserved populations. Additionally, one article (Dunbar & Reed, 1999) examined children living in poverty and another explored occupational therapy services to support older adults who were precariously housed (Merryman & Synovec, 2020). Two articles focussed solely on woman and disability (Eklund & Erlandsson, 2014; Gonzalez *et al*., 2015), and one article (Naidoo *et al.,* 2017) identified the need for occupational therapists in South Africa to take on advocacy roles that facilitate social inclusion and enhance access to resources for persons with a disability. Oliveira and Ferigato (2019) sought to understand how Brazilian occupational therapists working in primary care can support women who have experienced domestic and family violence.

A number of articles published in the United States discussed issues of access, which were linked to the broader health system and the current lack of funding mechanisms to support occupational therapy in primary care (Dahl-Popolzio *et al*., 2017; Jordan, 2019; AOTA, 2020). One article in the Canadian context (Donnelly *et al*., 2014) highlighted issues related to access and occupational therapy in primary care.

## Discussion

This paper is the first to provide a review of the role of occupational therapy in primary care including assessment and interventions in both research and non-research literature. The scoping review highlights that primary care is a relatively new practice setting for occupational therapy, with just under half of the papers being non-research and largely offering a visionary description of the potential roles of occupational therapy in primary care. However, the evidence specific to occupational therapy in primary care has doubled in the past five years with 8 of the 14 RCT’s published since 2018. The increasing attention demonstrates the expansion of this role in Canada, the United States, Europe, and Australia. Primary care occupational therapy is an area of tremendous research and practice opportunity.

The review emphasizes that occupational therapists working in primary care provide services aimed at promoting patients’ engagement in occupations, by addressing the person, the occupations in which they engage, and the home and community environments for individuals of all ages. Although occupational therapy may provide assessment and interventions to children and youth in primary care, the predominant focus in the literature is on adult and older adult populations, largely due to the emphasis on managing chronic conditions in adults and supporting older adults to live independently in their homes. Occupational therapists assessed and treated physical, cognitive and affective components of patients presenting to primary care settings. We identified a total of 181 unique interventions in the research literature highlighting both the depth and the breadth of the profession in primary care. This comprehensive approach to patient care is in keeping with generalist primary care practice, which has been described elsewhere in the literature (Devereaux & Walker,^1995^; Donnelly *et al*., 2014, 2016).

The most frequently identified primary care occupational therapy interventions were chronic disease management (*n* = 34)/self-management (*n* = 28), referring to/advocating for/coordinating/linking to and navigating community services (*n* = 36 articles), health promotion (*n* = 30) and falls prevention (*n* = 27). Health promotion is not so much a specific role, but rather a framework that identifies different levels at which occupational therapists can intervene to support patients in primary care. Wilcock (2006: 313) statds that “an occupation-focussed approach to health promotion involves enabling people to increase control over, and to improve, their health and that this can be attained through doing (occupations)”. Wilcock and Hocking (2015) present four levels of health-promoting occupational therapy that are reflective of the assessments and interventions identified in this scoping review. Level one is directed to the general public, focussing on preventing health-damaging behaviours and illness. The results at this level would include such roles as developmental screening, falls risk assessments and prevention, injury prevention, driver screening, healthy eating and weight management. Level two includes individuals who currently experience health issues to influence behaviour change and to slow down or prevent further disease and disability. A large number of the roles identified in this review fall into this level. Included here are lifestyle modifications, promotion of healthy habits and routines, chronic disease self-management activities including joint protection, energy conservation, home exercises, counselling, pain management and ergonomics. The third level addresses those individuals with chronic disease and disability and aims to maintain health and well-being. Occupational therapists most often work with this population and a range of roles fit here including prescription of assistive technology, home modifications, supporting access to disability supports and benefits, transportation, mobility interventions, caregiver support and education, facilitating return to work and supporting children’s performance at school. The final level is directed towards end-of-life care and supporting quality of life. While there were fewer roles identified for this final level, there was a clear identification of the role for occupational therapy to offer end-of-life care and support families and caregivers, particularly in the non-research literature.

Occupation-focussed interventions were the most frequently identified overall, which highlights the strong emphasis placed on supporting healthy daily activities and routines. In primary care, there is a growing focus on lifestyle medicine, whose aim is to support individuals to adopt behaviours to improve health and quality of life (Bourbeau *et al*., 2016; Rash *et al*., 2016). A number of barriers have been reported that limit primary care in providing lifestyle intervention including low confidence of providers, lack of time, belief about and knowledge of lifestyle interventions (Lianov & Johnston, 2010; Bouma *et al*., 2019). An interprofessional team has been recommended to enhance lifestyle medicine and a strong argument could be made that occupational therapy is ideally suited to support lifestyle medicine; focussing on occupation and understanding the impact of person-level factors on activities and routines within the context of their environments (Moll *et al*., 2015; AOTA, 2020).

This scoping review shed some light on occupational therapists’ work with at risk populations in primary care and health equity issues. Occupational therapists’ have a long history of working with underserved populations to redress inequities in health, valuing equity principles as part of their practice and seeking to enable a just society (Townsend & Wilcock, 2004; Restall *et al*., 2018). Occupational therapist, however, have also experienced challenges applying these principles across the health care system (Gerlach, 2015;Restall *et al*., 2018). The results of this review point to the fact occupational therapists in primary care are working to connect people to services across health and social systems and are well-placed to address social determinants of health and health inequities. Moving forward occupational therapists need to consider how they can and moving beyond traditional individual interventions in primary care settings and consider adopting human right approaches as suggested by Restall and colleagues (2018) to support people and communities.

Much of the early literature used to support the role of occupational therapy in primary care drew on occupational therapy research that could inform the role, with relevant populations, but was not directly studied in a primary care context. Many recent non-research papers that call for occupational therapy in primary care still largely rely on evidence gathered in non-primary care settings. For those people, who have disability and multiple conditions, the evidence about the effectiveness of occupational therapy interventions may be transferable to delivery in a primary care context, because the interventions themselves may be very similar regardless of the delivery site. However, it must be understood that primary care is a unique setting and what might work in other practice settings may not work in primary care.

One of the key limitations of the scoping review is the discrepancy in how primary care is defined and understood in occupational therapy, making it difficult, at times, to determine the specific setting in which the study was conducted. This lack of clarity within the profession could have led to missed research. Moving forward, researchers need to be clear in the description of the primary care setting and provide context regarding the health care models or services. The authors of this scoping review did not evaluate the strength of the research evidence in relation to the role and contributions of occupational therapists in primary care. While these are not part of the original scoping review methodology, they could have provided additional insights into the findings.

In conclusion, our review has demonstrated a breadth of contributions that occupational therapists are making in primary care with a role that is focussed on understanding how individuals are engaging in everyday activities and supporting them to participate in their communities through interventions targeted at building or adapting individual capacity, engaging in occupations and ensuring safe and accessible environments. Primary care is a relatively new practice setting for occupational therapists and offers a unique opportunity for the profession to draw on a health promotion lens, which the scoping review has demonstrated.

Primary care has almost exclusively been physician-based, where a biomedical model is the dominant practice framework. With the changing practice demographics, occupational therapists can bring their health promotion lens and unique understanding of everyday activities and function to primary care teams to support physicians and the broader team meet the more complex health and social needs seen in primary care. As the World Health Organization’s ‘Rehabilitation 2030 Initiative’ draws attention to the need to strengthen access to rehabilitation across health system, it is clear there is a need across the globe for ongoing efforts to ensure solidification and further integration of occupational therapy in primary care contexts.

## Supplementary material

For supplementary material accompanying this paper visit http://doi.org/10.1017/S1463423622000123. click here to view supplementary material

## References

[ref1] American Occupational Therapy Association (2014) Primary care. In *AOTA position statement: Occupational therapy in primary care*. American Occupational Therapy Association.

[ref2] American Occupational Therapy Association (2020) Role of occupational therapy in primary care. American Journal of Occupational Therapy, 74 (Suppl 3), 7413410040p1–7413419949p16. 10.5014/ajot.2020.74S3001 34935898

[ref3] Arksey, H. and O’Malley, L. (2005) Scoping studies: Towards a methodological framework. International Journal of Social Research Methodology, 8, 19–32. 10.1080/1364557032000119616

[ref4] Ashcroft, R. , Donnelly, C. , Dancey, M. , Gill, S. , Lam, S. , Kourgiantakis, T. , … Brown, J. B. (2021). Primary care teams’ experiences of delivering mental health care during the COVID-19 pandemic: a qualitative study. BMC Family Practice, 22, 1–12. 34210284 10.1186/s12875-021-01496-8 PMC8248293

[ref5] Ashcroft, R. , Donnelly, C. , Gill, S. , Dancey, M. , Lam, S. , Grill, A. K. and Mehta, K. (2021). The delivery of patient care in Ontario’s family health teams during the first wave of the COVID-19 pandemic. Healthcare Policy, 17, 72. 34895411 10.12927/hcpol.2021.26656 PMC8665725

[ref6] Baaijen, R. , Bolt, M. , Ikking, T. and Saenger, S. (2016) *Position paper: Occupational therapy and primary care*. COTEC Position Paper.

[ref7] Bauer, M. and O’Neill, C. (2012) Occupational therapy in primary health: “Right care, at the right time, in the right place. Occupational Therapy Now, 14, 5–6.

[ref8] Bergin, M. and Keegan, F. (2016) Reflecting our scope: Exploring the use of a theory driven initial assessment tool in primary care occupational therapy practice with children. The Irish Journal of Occupational Therapy, 44, 3–9.

[ref9] Bernstein, K. , Manning, D. and Julian, R. (2016) Multidisciplinary teams and obesity: Role of the modern patient-centered medical home. Primary Care: Clinics in Office Practice, 43, 53–59. 26896199 10.1016/j.pop.2015.08.010

[ref10] Blachman, N. L. and Blaum, C. S. (2016) Integrating care across disciplines. Clinics in Geriatric Medicine, 32, 373–383. 27113153 10.1016/j.cger.2016.01.010

[ref11] Boakye, O. , Birney, A. , Suter, E. , Phillips, L. A. and Suen, V. Y. M. (2016) Scope of practice review: Providers for triage and assessment of spine-related disorders. Journal of Multidisciplinary Healthcare, 9, 227. 10.2147/JMDH.S97590 27274267 PMC4869847

[ref12] Bolling, C. and Mesquita, A. (2012) Work process and challenges in the multidisciplinary support team for family health strategy (NASF). Journal of Science and Medicine in Sport, 15, S158.

[ref13] Bolt, M. , Ikking, T. , Baaijen, R. and Saenger, S. (2019) Scoping review: occupational therapy interventions in primary care. Primary Health Care Research & Development, 20. 10.1017/S146342361800049X PMC6476367 32799994

[ref14] Bouma, A. J. , van Wilgen, P. , Baarveld, F. , Lemmink, K. A. , Diercks, R. L. and Dijkstra, A. (2019) A cross-sectional analysis of motivation and decision making in referrals to lifestyle interventions by primary care general practitioners: a call for guidance. American Journal of Lifestyle Medicine, 13, 301–311. 31105494 10.1177/1559827617694594 PMC6506977

[ref15] Bourbeau, J. , Lavoie, K. L. , Sedeno, M. , De Sousa, D. , Erzen, D. , Hamilton, A. , Maltais, F. , Troosters, T. and Leidy, N. (2016) Behaviour-change intervention in a multicentre, randomised, placebo controlled COPD study: Methodological considerations and implementation. BMJ Open, 6, e010109. 10.1136/bmjopen-2015-010109 PMC4823464 27044576

[ref16] Brandis, S. J. and Tuite, A. T. (2001) Falls prevention: Partnering occupational therapy and general practitioners. Australian Health Review, 24, 37–42. 10.1071/AH010037 11357740

[ref17] Brown, C. and Diamond-Burchuk, L. (2013) Occupational therapists providing interprofessional education and enhancing health within a teaching primary care centre. Occupational Therapy Now.

[ref18] Brown, CL , Leclair, LL , Fricke, M and Wener, P. (2021) Discrepancy between attitudes and behaviors of family medicine residents towards interprofessional collaborative practice: a mixed methods study. Journal of Interprofessional Education & Practice, 23, 100374. 10.1016/j.xjep.2020.100374

[ref19] Canadian Association of Occupational Therapists (2013) Occupational Therapy in Primary Care. In CAOT position statement. Canadian Association of Occupational Therapists.

[ref20] Carlsson, L. , Englund, L. , Hallqvist, J. and Wallman, T. (2013) Early multidisciplinary assessment was associated with longer periods of sick leave: A randomized controlled trial in a primary health care centre. Scandinavian Journal of Primary Health Care, 31, 141–146. 10.3109/02813432.2013.811943 23909930 PMC3750435

[ref21] Cassidy, T. B. , Richards, L. G. and Eakman, A. M. (2017) Feasibility of a lifestyle Redesign®-inspired intervention for well older adults. American Occupational Therapy Association, 71, p1–7104190050. 10.5014/ajot.2017.024430 28691677

[ref22] Clemson, L. , Donaldson, A. , Hill, K. and Day, L. (2014) Implementing person-environment approaches to prevent falls: A qualitative inquiry in applying the Westmead approach to occupational therapy home visits. Australian Occupational Therapy Journal, 61, 325–334. 10.1111/1440-1630.12132 24825447

[ref23] College of Family Physicians of Canada (2019) A new vision for Canada: Family Practice—The Patient’s Medical Home 2019. College of Family Physicians of Canada. Author.

[ref24] Connolly, D. (2016) Occupational therapy practice in primary care: Responding to changing health priorities and needs in Ireland. The Irish Journal of Occupational Therapy, 44.

[ref25] Connolly, D. , Anderson, M. , Colgan, M. , Montgomery, J. , Clarke, J. and Kinsella, M. (2018) The impact of a primary care stress management and wellbeing programme (RENEW) on occupational participation: A pilot study. British Journal of Occupational Therapy, 1–10. 10.1177/0308022618793323

[ref26] Cook, S. (2003) Generic and specialist interventions for people with severe mental health problems: Can interventions be categorised? British Journal of Occupational Therapy, 66, 17–24. 10.1177/030802260306600104

[ref27] Cook, S. and Howe, A. (2003) Engaging people with enduring psychotic conditions in primary mental health care and occupational therapy. British Journal of Occupational Therapy, 66, 236–246. 10.1177/030802260306600602

[ref28] Cook, S. , Howe, A. and Veal, J. (2004) A different ball game altogether: Staff views on a primary mental healthcare service. Primary Care Mental Health, 2, 77–89.

[ref29] Crawford-White, J. (1996) Are primary health-care occupational therapists specialists or generalists? British Journal of Therapy and Rehabilitation, 3, 373–379. 10.12968/bjtr.1996.3.7.14801

[ref30] Cunningham, R and Valasek, S. (2019) Occupational therapy interventions for urinary dysfunction in primary care: A case series. AJOT: American Journal of Occupational Therapy, 73, 7305185040p1–7305185040p8. 10.5014/ajot.2019.038356 31484023

[ref31] Dahl-Popolizio, S. , Manson, L. , Muir, S. and Rogers, O. (2016) Enhancing the value of integrated primary care: The role of occupational therapy. Families, Systems & Health: The Journal of Collaborative Family Care, 34, 270–280. 10.1037/fsh0000208 27441739

[ref32] Dahl-Popolizio, S. (2017) Interprofessional primary care: the value of occupational therapy. Open Journal of Occupational Therapy, 5, 11. 10.15453/2168-6408.1363

[ref33] Day, L. , Donaldson, A. , Thompson, C. and Thomas, M. (2014) Integrating proven falls prevention interventions into government programs. Australian and New Zealand Journal of Public Health, 38, 122–127. 10.1111/1753-6405.12140 24690049

[ref34] Devereaux, E. B. and Walker, R. B. (1995) The role of occupational therapy in primary health care. American Journal of Occupational Therapy, 49, 391–396. 10.5014/ajot.49.5.391 7598152

[ref35] Donnelly, C. , Ashcroft, R. , Bobbette, N. , Mills, C. , Mofina, A. , Tran, T. , … Miller, J. (2021). Interprofessional primary care during COVID-19: A survey of the provider perspective. BMC Family Practice, 22, 1–12. 33535973 10.1186/s12875-020-01366-9 PMC7857097

[ref36] Donnelly, C. , Brenchley, C. , Crawford, C. and Letts, L. (2013) The integration of occupational therapy into primary care: A multiple case study design. BMC Family Practice, 14, 60. 10.1186/1471-2296-14-60 23679667 PMC3663696

[ref37] Donnelly, C. A. , Brenchley, C. L. , Crawford, C. N. and Letts, L. J. (2014) The emerging role of occupational therapy in primary care. Canadian Journal of Occupational Therapy, 81, 51–61. 10.1177/0008417414520683 24783488

[ref38] Donnelly, C. and Letts, L. (2013) 10 tips to integrate occupational therapy in primary care teams. Occupational Therapy Now, 15, 7–8.

[ref39] Donnelly, C. A. , Leclair, L. L. , Wener, P. F. , Hand, C. L. and Letts, L. J. (2016) Occupational therapy in primary care: Results from a national survey. Canadian Journal of Occupational Therapy, 83, 135–142. 10.1177/0008417416637186 27074910

[ref40] Donnelly, C. , O’Neill, C. , Bauer, M. and Letts, L. (2017) Canadian Occupational Performance Measure (COPM) in primary care: a profile of practice. AJOT: American Journal of Occupational Therapy, 71, 7106265010p1–7106265010p8. 10.5014/ajot.2017.020008 29135432

[ref41] Drummond, A. , Coole, C. , Nouri, F. , Ablewhite, J. and Smyth, G. (2020) Using occupational therapists in vocational clinics in primary care: a feasibility study. BMC Family Practice, 21, 268. 10.1186/s12875-020-01340-5 33308145 PMC7734822

[ref42] Dunbar, S. B. and Reed, C. N. (1999) A developmental screening program in primary health care: Meeting the challenges of children in low-income families. Infant-Toddler Intervention: The Transdisciplinary Journal, 9, 195–202.

[ref43] Eichler, J. and Royeen, L. (2016) Occupational therapy in the primary health care clinic: Experiences of two clinicians. Families, Systems & Health, 34, 289–292. 10.1037/fsh0000226 27632545

[ref44] Eklund, M. and Erlandsson, L. K. (2014) Women’s perceptions of everyday occupations: Outcomes of the Redesigning Daily Occupations (ReDO) programme. Scandinavian Journal of Occupational Therapy, 21, 359–367. 10.3109/11038128.2014.922611 24878142

[ref45] Fong, K. N. (2008) Occupational therapy in primary health care: A new area for involvement and contributions in the new health care system in Hong Kong. Hong Kong Journal of Occupational Therapy, 18, i–ii.

[ref46] Fox, J , Erlandsson, LK and Shiel, A. (2021) A feasibility study of the Redesigning Daily Occupations (ReDOTM-10) programme in an Irish context. Scandinavian Journal of Occupational Therapy, 1–15. 10.1080/11038128.2021.1882561 33556290

[ref47] Frenchman, K. (2014) The health promoting role of occupational therapy in primary health care: A reflection and emergent vision. New Zealand Journal of Occupational Therapy, 61, 64.

[ref48] Fritz, H , Hu, YL , Tarraf, W and Patel, P. (2019) Feasibility of a habit formation intervention to delay frailty progression among older African Americans: a pilot study. The Gerontologist, 60, 1353–1363. 10.1093/geront/gnz143 31688909

[ref49] Fry, D. , Fox, B. and Donnelly, C. (2013) Traveling a New Road: A driving cessation group in primary care. Occupational Therapy Now, 15, 25–26.

[ref50] Gallagher, A. M. , Lyons, B. , Houston, C. and Cummins, M. (2016) Exploring the current and potential role for occupational therapists in managing depression in primary care settings: Perspectives on occupational therapists in Ireland. The Irish Journal of Occupational Therapy, 44, 10–18.

[ref51] Garvey, J. , Connolly, D. , Boland, F. and Smith, S. M. (2015) OPTIMAL, an occupational therapy led self-management support programme for people with multimorbidity in primary care: A randomized controlled trial. BMC Family Practice, 16, 59. 10.1186/s12875-015-0267-0 25962515 PMC4438474

[ref52] Gaynord, B. (1996) Case studies: Primary care occupational therapy: Paula and Jack. British Journal of Therapy and Rehabilitation, 3, 386–388. 10.12968/bjtr.1996.3.7.14803

[ref53] Gerlach, A. J. (2015) Sharpening our critical edge: Occupational therapy in the context of marginalized populations. Canadian Journal of Occupational Therapy, 82, 245–253. 10.1177/0008417415571730 26502020

[ref54] Glennon, T. J. and Meriano, C. (2014) Collaboration in primary care under the ACA: What is the occupational therapy role? Administration & Management Special Interest Section Quarterly, 30, 1–4.

[ref55] Gonzalez, J. G. , del Teso Rubio, M. D. M. , Paniagua, C. N. W. , Criado-Alvarez, J. J. and Holgado, J. S. (2015) Symptomatic pain and fibromyalgia treatment through multidisciplinary approach for primary care. Reumatología Clínica (English Edition), 11, 22–26. 10.1016/j.reumae.2014.03.019 24837647

[ref56] Goossen, B. (2013) Reflecting on key influences shaping occupational therapy services in a Saskatoon primary health care setting. Occupational Therapy Now, 15, 27–28.

[ref57] Government of Manitoba. (2012) *Primary care interprofessional team toolkit*. Manitoba Health, Seniors and Active Living.

[ref58] Government of Ontario (2005) Family Health Teams- Guide to Interdisciplinary Team Roles and Responsibilities. Ministry of Health and Long-Term Care.

[ref59] Gudkovs, J. (2011) Intentional teams in rural health care: A preliminary report. Australasian Psychiatry, 19 (Suppl 1), S98–S101. 10.3109/10398562.2011.583063 21878032

[ref60] Gustavsson, C. , Nordlander, J. and Söderlund, A. (2018) Activity and life-role targeting rehabilitation for persistent pain: Feasibility of an intervention in primary healthcare. European Journal of Physiotherapy, 20, 141–151. 10.1080/21679169.2018.1426784

[ref61] Halle, A. D. , Mroz, T. M. , Fogelberg, D. J. and Leland, N. E. (2018) Occupational therapy and primary care: updates and trends’, The American Journal of Occupational Therapy, 72, 1–6. 10.5014/ajot.2018.723001 PMC5915228 29689169

[ref62] Hand, C. L. , Letts, L. J. and von Zweck, C. M. (2011) An agenda for occupational therapy’s contribution to collaborative chronic disease research. Canadian Journal of Occupational Therapy, 78, 147–155. 10.2182/cjot.2011.78.3.2 21699008

[ref63] Hansson, E. E. , Håkansson, E. , Raushed, A. and Håkansson, A. (2009) Multidisciplinary program for stress-related disease in primary health care. Journal of Multidisciplinary Healthcare, 2, 61. 10.2147/JMDH.S5298 21197348 PMC3004555

[ref64] Hart, E. C. and Parsons, H. (2015) Occupational therapy: Cost-effective solutions for a changing health system. American Occupational Therapy Association, Inc.

[ref65] Holmqvist, K. , Ivarsson, A. B. and Holmefur, M. (2014) Occupational therapist practice patterns in relation to clients with cognitive impairment following acquired brain injury. Brain Injury, 28, 1365–1373. 10.3109/02699052.2014.919529 24911987

[ref66] Howey, M. , Angelucci, T. , Johnston, D. and Townsend, E. A. (2003) Occupation-based program development in primary health care. Occupational Therapy Now, 11, 4–7.

[ref67] Hutchison, B. , Levesque, J. F. , Strumpf, E. and Coyle, N. (2011) Primary health care in Canada: Systems in motion. The Milbank Quarterly, 89, 256–288. 10.1111/j.1468-0009.2011.00628.x 21676023 PMC3142339

[ref68] Jaakkimainen, L. , Upshur, R. , Klein-Geltink, J. , Leong, A. , Maaten, S. , Schultz, S. and Wang, L. (2006) Primary Care in Ontario: ICES atlas. Toronto: Institute for Clinical Evaluative Sciences.

[ref69] Johansson, A. and Björklund, A. (2006) Occupational adaptation or well-tried, professional experience in rehabilitation of the disabled elderly at home. Activities, Adaptation and Aging, 30, (1), 1–21. 10.1300/J016v30n01_01

[ref70] Johansson, A. and Björklund, A. (2016) The impact of occupational therapy and lifestyle interventions on older persons’ health, well-being, and occupational adaptation: A mixed-design study. Scandinavian Journal of Occupational Therapy, 23, 207–219. 10.3109/11038128.2015.1093544 26442837

[ref71] Johansson, E. , Jonsson, H. , Dahlberg, R. and Patomella, A. H. (2018) The efficacy of a multifactorial falls-prevention programme, implemented in primary health care. British Journal of Occupational Therapy, 81, 474–481. 10.1177/0308022618756303

[ref72] Jordan, K. (2019) Occupational therapy in primary care: positioned and prepared to be a vital part of the team. AJOT: American Journal of Occupational Therapy, 73, 7305170010p1–7305170010p6. 10.5014/ajot.2019.735002 31484019

[ref73] Jordan, K. (2020) A provider – patient relationship: the critical first step of smoking cessation. Journal of General Internal Medicine, 35, 10–11. 10.1007/s11606-019-05276-0 31720963 PMC6957666

[ref74] Killian, C. , Fisher, G. and Muir, S. (2015) Primary care: A new context for the scholarship of practice model. Occupational Therapy in Health Care, 29, 383–396. 10.3109/07380577.2015.1050713 26115142

[ref75] King, D. K. , Estabrooks, P. A. , Strycker, L. A. , Toobert, D. J. , Bull, S. S. and Glasgow, R. E. (2006) Outcomes of a multifaceted physical activity regimen as part of a diabetes self-management intervention. Annals of Behavioral Medicine, 31, 128–137. 10.1207/s15324796abm3102_4 16542127

[ref76] Kowalski, L. and Krusen, N. E. (2021) Lung cancer screening policy in Alaska and occupational therapy. AJOT: American Journal of Occupational Therapy, 75, 7503090010, 10.5014/ajot.2021.048231 34781340

[ref77] Koverman, B. , Royeen, L. and Stoykov, M. (2017) Occupational therapy in primary care: Structures and processes that support integration. The Open Journal of Occupational Therapy, 5. 10.15453/2168-6408.1376

[ref78] Lamarche, L. , Bailey, K. A. , Awan, A. , Risdon, C. , Pauw, G. and Vinoski Thomas, E. (2020) Exploring primary care providers’ understandings of body image in patient care. Body Image, 35, 161–170. 10.1016/j.bodyim.2020.09.001 33049456

[ref79] Lamb, S. E. , Lall, R. , Hansen, Z. , Castelnuovo, E. , Withers, E. J. , Nichols, V. , Griffiths, F. , Potter, R. , Szczepura, A. and Underwood, M. (2010) A multicentred randomised controlled trial of a primary care-based cognitive behavioural programme for low back pain: The Back Skills Training (BeST) trial. Health Technology Assessment, 14, 1–281. 10.3310/hta14410 20807469

[ref80] Lamb, A. J. and Metzler, C. A. (2014) Defining the value of occupational therapy: A health policy lens on research and practice. American Journal of Occupational Therapy, 68, 9–14. 10.5014/ajot.2014.681001 24367949

[ref81] Lambert, R. A. , Harvey, I. and Poland, F. (2007) A pragmatic, unblinded randomised controlled trial comparing an occupational therapy-led lifestyle approach and routine GP care for panic disorder treatment in primary care. Journal of Affective Disorders, 99, (1), 63–71. 10.1016/j.jad.2006.08.026 17014912

[ref82] Lapointe, J. , James, D. and Craik, J. (2013) Occupational therapy services for people living with HIV: A case of service delivery in a primary health care setting. Occupational Therapy Now, 15, 22–24.

[ref83] Law, M. , Steinwender, S. and Leclair, L. (1998) Occupation, health and well-being. Canadian Journal of Occupational Therapy, 65, 81–91.

[ref84] Leclair, L. (2013) Occupational therapists in primary health care and primary care: Important contributors to the interprofessional team. Occupational Therapy Now, 15, 3–4.

[ref85] Leclair, L. , Restall, G. , Edwards, J. , Cooper, J. , Stern, M. , Soltys, P. and Sapacz, R. (2005) Occupational therapists and primary health care. Winnipeg: Manitoba Society of Occupational Therapists.

[ref86] Letts, L. J. (2011) Optimal positioning of occupational therapy. Canadian Journal of Occupational Therapy, 78, 209–217. 10.2182/cjot.2011.78.4.2 22043552

[ref87] Levac, D. , Colquhoun, H. and O’Brien, K. K. (2010) Scoping studies: Advancing the methodology. Implementation Science, 5, 69. 10.1186/1748-5908-5-69 20854677 PMC2954944

[ref88] Lianov, L. and Johnson, M. (2010). Physician competencies for prescribing lifestyle medicine. JAMA, 304, 202–203. 20628134 10.1001/jama.2010.903

[ref89] Locas, V , Préfontaine, C , Veillette, N and Vachon, B. (2019) Integration of occupational therapists into family medicine groups: physicians’ perspectives. British Journal of Occupational Therapy, 83, 458–468. 10.1177/0308022619883481

[ref90] Kringos, D.S. , Boerma, W.G. , Hutchinson, A. and Saltman, R.B. (2015) Building primary care in a changing Europe. WHO Regional Office for Europe. 29035488

[ref91] Mackenzie, L. , Clemson, L. and Roberts, C. (2013) Occupational therapists partnering with general practitioners to prevent falls: Seizing opportunities in primary health care. Australian Occupational Therapy Journal, 60, 66–70. 10.1111/1440-1630.12030 23414191

[ref92] Mackenzie, L. , Lovarini, M. , Price, T. , Clemson, L. , Tan, A. and O’Connor, C. (2018) An evaluation of the fall prevention practice of community-based occupational therapists working in primary care. British Journal of Occupational Therapy, 81, 463–473. 10.1177/0308022618764798

[ref93] Mårtensson, B. F. L. and Marklund, B. (1999) Evaluation of a biopsychosocial rehabilitation programme in primary healthcare for chronic pain patients. Scandinavian Journal of Occupational Therapy, 6, 157–165. 10.1080/110381299443636

[ref94] Marval, R. (2018) Community-tailored occupational therapy in primary health care to promote individual and community health. Occupational Therapy Now. https://caot.ca/document/5751/Community-tailored%20occupational%20therapy%20in%20primary%20health.pdf

[ref95] McColl, M. A. and Dickenson, J. (2009) Inter-professional primary health care: Assembling the pieces. A framework to build your practice in primary health care. Canadian Association of Occupational Therapists.

[ref96] McColl, M. A. , Shortt, S. , Godwin, M. , Smith, K. , Rowe, K. , O’Brien, P. and Donnelly, C. (2009) Models for integrating rehabilitation and primary care: A scoping study. Archives of Physical Medicine and Rehabilitation, 90, 1523–1531. 10.1016/j.apmr.2009.03.017 19735780

[ref97] McGrath, M. and O’Callaghan, C. (2014) Occupational therapy and dementia care: A survey of practice in the Republic of Ireland. Australian Occupational Therapy Journal, 61, 92–101. 10.1111/1440-1630.12081 24689920

[ref98] Mercer, S. W. , Smith, S. M. , Wyke, S. , O’Dowd, T. and Watt, G. C. M. (2009) Multimorbidity in primary care: Developing the research agenda. Family Practice, 26, 79–80. 10.1093/fampra/cmp020 19287000

[ref99] Merryman, M. B. and Synovec, C. E. (2020) Integrated care: provider referrer perceptions of occupational therapy services for homeless adults in an integrated primary care setting. Work, 65, 321–330. 10.3233/WOR-203084 32007976

[ref100] Metzler, C. A. , Hartmann, K. D. and Lowenthal, L. A. (2012) Defining primary care: Envisioning the roles of occupational therapy. American Journal of Occupational Therapy, 66, 266–270. 10.5014/ajot.2010.663001 22549590

[ref101] Middlebrook, S. and Mackenzie, L. (2012) The enhanced primary care program and falls prevention: Perceptions of private occupational therapists and physiotherapists. Australasian Journal on Ageing, 31, 72–77. 10.1111/j.1741-6612.2011.00527.x 22676164

[ref102] Mirza, M. , Gecht-Silver, M. , Keating, E. , Krischer, A. , Kim, H. and Kottorp, A. (2020) Feasibility and preliminary efficacy of an occupational therapy intervention for older adults with chronic conditions in a primary care clinic. The American Journal of Occupational Therapy, 74, 7405205030p1–7405205030p13. 10.5014/ajot.2020.039842 PMC7430728 32804621

[ref103] Moll, S.E. , Gewurtz, R.E. , Krupa, T.M. , Law, M.C. , Lariviere, N. and Levasseur, M. (2015) “Do-Live-Well”: A Canadian framework for promoting occupation, health, and well-being: «Vivez-Bien-Votre Vie»: un cadre de référence canadien pour promouvoir l’occupation, la santé et le bien-être. Canadian Journal of Occupational Therapy, 82, 9–23. 10.1177/0008417414545981 25803944

[ref104] Muir, S. (2012) Occupational therapy in primary health care: We should be there. American Journal of Occupational Therapy, 66, 506–510. 10.5014/ajot.2012.665001 22917116

[ref105] Muir, S. , Henderson-Kalb, J. , Eichler, J. , Serfas, K. and Jennison, C. (2014) Occupational therapy in primary care: An emerging area of practice. OT Practice, 19.

[ref106] Murphy-Turliuk, A. (2013) Ontario occupational therapists’ experience of integrating into family health teams. Occupational Therapy Now, 15, 9–11.

[ref107] Murphy, A. D. , Griffith, V. M. , Berkeridge, T. , Mroz, T. M. and Jirikowic, T. L. (2017) Primary care for underserved populations: Navigating policy to incorporate occupational therapy into federally qualified health centers. American Journal of Occupational Therapy, 71, 7102090010p1–7102090010p5. 10.5014/ajot.2017.712001 28218582

[ref108] Naidoo, D. , Van Wyk, J. and Joubert, R. (2017) Community stakeholders’ perspectives on the role of occupational therapy in primary healthcare: Implications for practice. African Journal of Disability, 6, 1–12. 10.4102/ajod.v6i0.255 PMC5502470 28730063

[ref109] Naumann, D. N. (2011) Occupational therapists as knowledge brokers: Leading knowledge translation in primary care. Occupational Therapy Now, 15, 29–31.

[ref110] NICE (2008) Mental wellbeing in over 65s: Occupational therapy and physical activity interventions. National Institute for Health and Care Excellence. Public Health Guideline.

[ref111] Norberg, E. B. , Löfgren, B. , Boman, K. , Wennberg, P. and Brännström, M. (2017) A client-centred programme focusing energy conservation for people with heart failure. Scandinavian Journal of Occupational Therapy, 24, 455–467. 10.1080/11038128.2016.1272631 28052703

[ref112] Oliveira, M. T. and Ferigato, S. H. (2019) ‘The attention to women victims of domestic and family violence: Care technologies of occupational therapy in basic health care [A atenção às mulheres vítimas de violência doméstica e familiar: a construção de tecnologias de cuidado da terapia ocupacional na atenção básica em saúde]. Cadernos de Terapia Ocupacional da UFSCar, 27, 508–521. 10.4322/2526-8910.ctoAO1729

[ref113] Olsson, A. , Erlandsson, L. K. and Hakansson, C. (2020) The occupation-based intervention REDO-10: Long term impact on work ability for women at risk for or on sick leave. Scandinavian Journal of Occupational Therapy, 27, 47–55. https://doi.org/10.1080.11038128.2019.1614215 31099284 10.1080/11038128.2019.1614215

[ref114] O’Toole, L. , Connolly, D. and Smith, S. (2013) Impact of an occupation-based self-management programme on chronic disease management. Australian Occupational Therapy Journal, 60, 30–38. 10.1111/1440-1630.12008 23414187

[ref115] O’Toole, L. , Connolly, D. , Boland, F. and Smith, S. M. (2021) Effect of the OPTIMAL programme on self-management of multimorbidity in primary care: A randomised control trial. The British Journal of General Practice: The Journal of the Royal College of General Practitioners, 71, e303–e311. 10.3399/bjgp20X714185 33685920 PMC7959668

[ref117] Ontario Society of Occupational Therapists. (n.d.). Occupational therapists in primary health care- Working to support your family health team. In Ontario Society of Occupational Therapists. Web Resource.

[ref118] Peranich, L. , Reynolds, K. B. , O’Brien, S. , Bosch, J. and Cranfill, T. (2010) The roles of occupational therapy, physical therapy, and speech/language pathology in primary care. Journal for Nurse Practitioners, 6, 36–43. 10.1016/j.nurpra.2009.08.021

[ref119] Peters, M. D. , Godfrey, C. M. , Khalil, H. , McInerney, P. , Parker, D. and Soares, C. B. 2015. Guidance for conducting systematic scoping reviews. International Journal of Evidence-Based Healthcare, 13, 141–146. 10.1097/XEB.0000000000000050 26134548

[ref120] Phelan, E. A. , Mahoney, J. E. , Voit, J. C. and Stevens, J. A. (2015) Assessment and management of fall risk in primary care settings. Medical Clinics of North America, 99, 281–293. 10.1016/j.mcna.2014.11.004 25700584 PMC4707663

[ref122] Pritchard, S. (2013) Enhancing primary care for persons with spinal cord injuries: The role of occupational therapy on an interdisciplinary team. Occupational Therapy Now, 15, 5–6.

[ref123] Public Health Agency of Canada (2017) How healthy are Canadians? A trend analysis of the health of Canadians from a healthy living and chronic disease perspective. Public Health Agency of Canada: Ottawa, ON. https://www.canada.ca/en/public-health/services/publications/healthy-living/how-healthy-canadians.html

[ref116] Pyatak, E. , King, M. , Vigen, C. L. P. , Salazar, E. , Diaz, J. , Niemiec, S. L. S. , Blanchard, J. , Jordan, K. , Banerjee, J. and Shukla, J. (2019) Addressing diabetes in primary care: Hybrid effectiveness-implementation study of lifestyle Redesign® occupational therapy. AJOT: American Journal of Occupational Therapy, 73, 7305185020p1–7305185020p12. https://doi.org/10.5014.ajot.2019.037317 10.5014/ajot.2019.037317 PMC9017643 31484021

[ref124] Rash, J. A. , Lavoie, K. L. , Sigal, R. J. , Campbell, D. J. T. , Manns, B. J. , Tonelli, M. and Campbell, T. S. (2016) The OPTIMIZE trial: Rationale and design of a randomized controlled trial of motivational enhancement therapy to improve adherence to statin medication. Contemporary Clinical Trials, 49, 47–56. 10.1016/j.cct.2016.06.001 27282119

[ref125] Restall, G. , Leclair, L. and Fricke, M. (2005) Integration of occupational therapy and physiotherapy services in primary health care in Winnipeg. School of Medical Rehabilitation, University of Manitoba.

[ref126] Restall, G. J. , MacLeod Schroeder, N. J. and Dubé, C. D. (2018) The equity lens for occupational therapy: A program development and evaluation tool. Canadian Journal of Occupational Therapy, 85, 185–195. 10.1177/0008417418756421 29972050

[ref127] Rexe, K. , Lammi, B. M. and von Zweck, C. (2013) Occupational therapy: Cost-effective solutions for changing health system needs. Healthcare Quarterly, 16, 69–75. 24863311

[ref128] Richardson, J. , Letts, L. , Chan, D. , Officer, A. , Wojkowski, S. , Oliver, D. , Moore, A. , McCarthy, L. , Price, D. and Kinzie, S. (2012) Monitoring physical functioning as the sixth vital sign: Evaluating patient and practice engagement in chronic illness care in a primary care setting – A quasi-experimental design. BMC Family Practice, 13, 29. 10.1186/1471-2296-13-29 22471378 PMC3355020

[ref129] Richardson, J. , Letts, L. , Chan, D. , Stratford, P. , Hand, C. , Price, D. , Hilts, L. , Coman, L. , Edwards, M. , Baptiste, S. and Law, M. (2010) Rehabilitation in a primary care setting for persons with chronic illness – A randomized controlled trial. Primary Health Care Research and Development, 11, 382–395. 10.1017/S1463423610000113

[ref130] Rovner, B. W. , Casten, R. J. , Piersol, C. V. , White, N. , Kelley, M. and Leiby, B. E. (2020) Improving glycemic control in African Americans with diabetes and mild cognitive impairment. Journal of the American Geriatrics Society, 68, 1015–1022. 10.1111/jgs.16339 32043561 PMC8311466

[ref131] Schepens Niemiec, S. L. , Vigen, C. L. P. , Martinez, J. , Blanchard, J. and Carlson, M. (2021) Long-term follow-up of a lifestyle intervention for late-midlife, rural-dwelling Latinos in primary care. American Journal of Occupational Therapy, 75, 7502205020p1–7502205020p11. 10.5014/ajot.2021.042861 PMC7929605 33657344

[ref132] Schepens Niemiec, S. L. , Blanchard, J. , Vigen, C. L. P. , Martínez, J. , Guzmán, L. , Fluke, M. and Carlson, M. (2019) A pilot study of the Vivir Mi VIda lifestyle intervention for rural dwelling late midlife Latinos: Study design and protocol. Occupational Therapy Journal of Research: Occupation, Participation and Health, 39, 5–13. 10.1177/1539449218762728 PMC6119523 29514544

[ref133] Schepens Niemiec, S. L. , Blanchard, J. , Vigen, C. L. P. , Martínez, J. , Guzmán, L. , Concha, A. , Fluke, M. and Carlson, M. (2018) Evaluation of ¡vivir Mi Vida! to improve health and wellness of rural-dwelling, late middle-aged Latino adults: Results of a feasibility and pilot study of a lifestyle intervention. Primary Health Care Research and Development, 19, 448–463. 10.1017/S1463423617000901 29729677 PMC6177291

[ref134] Society of Alberta Occupational Therapists (2016) The Role of Occupational Therapy (OT) in Primary Care. Society of Alberta Occupational Therapists.

[ref135] Somé, N. H. , Devlin, R. A. , Mehta, N. , Zaric, G. S. and Sarma, S. (2020) Team-based primary care practice and physician’s services: Evidence from Family Health Teams in Ontario, Canada. Social Science & Medicine, 264, 113310. 32877846 10.1016/j.socscimed.2020.113310

[ref136] Starfield, B. (1994) Is primary care essential? The Lancet, 344 (Suppl. 4-3), 1129–1133. 10.1016/S0140-6736(94)9063 7934497

[ref137] Stoffer-Marx, M. A. , Klinger, M. , Luschin, S. , Meriaux-Kratochvila, S. , Zettel-Tomenendal, M. , Nell-Duxneuner, V. , Zwerina, J. , Kjeken, I. , Hackl, M. , Uhlinger, S. , Woolf, A. , Redlich, K. , Smolen, J. S. and Stamm, T. A. (2018) Functional consultation and exercises improve grip strength in osteoarthritis of the hand – A randomised controlled trial. Arthritis Research & Therapy, 20, 253. 10.1186/s13075-018-1747-0 30413191 PMC6235228

[ref138] Sturesson, M. , Bylund, S. H. , Edlund, C. , Falkdal, A. H. and Bernspång, B. (2020) Collaboration in work ability assessment for sick-listed persons in primary healthcare. Work, 65, 409–420. 10.3233/WOR-203093 32007984

[ref139] Synovec, C. E. (2020) Evaluating cognitive impairment and its relation to function in a population of individuals who are homeless. Occupational Therapy in Mental Health, 36, 330–352. 10.1080/0164212X.2020.1838400

[ref140] Synovec, C. E. , Merryman, M. and Brusca, J. (2020) Occupational therapy in integrated primary care: addressing the needs of individuals experiencing homelessness. Open Journal of Occupational Therapy, 8, 1–14. 10.15453/2168-6408.1699 33552752

[ref141] Tinnelly, M. and Byrne, M. (2016) Primary care occupational therapy: Exploring the perceptions of therapists’ role and their current practice in Ireland. The Irish Journal of Occupational Therapy, 44, 23–31.

[ref142] Townsend, E. A. and Polatajko, H. J. (2013) Enabling occupation II: advancing an occupational therapy vision for health, well-being, and justice through occupation. Canadian Association of Occupational Therapists, 428 pp.

[ref143] Townsend, E. and Wilcock, A. A. (2004) Occupational justice and client centred practice: A dialogue in progress. Canadian Journal of Occupational Therapy, 71, 75–87. 10.1177/000841740407100203 15152723

[ref144] Tracy, C. S. , Bell, S. H. , Nickell, L. A. , Charles, J. and Upshur, R. E. G. (2013) The IMPACT clinic. Innovative model of interprofessional primary care for elderly patients with complex health care needs. Canadian Family Physician, 59, 148–155. PMC3596224 23486816

[ref145] Tran, T. , Donnelly, C. , Nalder, E. J. , Trothen, T. and Finlayson, M. (2020) Occupational therapist-led mindfulness-based stress reduction for older adults living with subjective cognitive decline or mild cognitive impairment in primary care: a feasibility randomised control trial protocol’, BMJ Open, 10, e035299. 10.1136/bmjopen-2019-035299 PMC7312340 32580984

[ref146] Trembath, F. , Dahl-Popolizio, S. , Vanwinkle, M. and Milligan, L. (2019) Retrospective analysis: most common diagnoses seen in a primary care clinic and corresponding occupational therapy interventions. Open Journal of Occupational Therapy, 7. 10.15453/2168-6408.1539

[ref147] Tse, S. , Penman, M. and Simms, G. (2003) Literature review: Occupational therapy and primary health care. New Zealand Journal of Occupational Therapy, 50, 17–23.

[ref148] Usher, R. , Payne, C. , Real, S. and Carey, L. (2021) Project ECHO: enhancing palliative care for primary care occupational therapists and physiotherapists in Ireland. Health & Social Care in the Community, n/a. 10.1111/hsc.13372 33991147

[ref149] Valasek, S. and Halle, A. (2018) Practicing in an established primary care setting: Practical tips and considerations. OT Practice, 23, 8–11. 10.7138/OTP.2018.2315.F1

[ref150] Villegas, N. L. (2016) Online course to expand occupational therapy practice: Education and implementation of occupational therapy in primary care. Doctoral dissertation, Boston University.

[ref151] Wallace, P. and Seidman, J. (2007) Improving population health and chronic disease management. In J. Dorland and M. A. McColl (Eds.), *Emerging approaches to chronic disease management in primary health care* (pp. 15–20). Queen’s Policy Studies Series.

[ref152] White, J. S. , Toto, P. , Skidmore, E. and Baker, N. (2020) Providing occupational therapy in a free primary care clinic. American Journal of Occupational Therapy, 35 (Suppl. 1), 26–32, 10.1080/13561820.2021.1981261

[ref153] Wilcock, A. (2006) An occupational perspectice of health. Thorofare, NJ: SLACK Inc.

[ref154] Wilcock, A. and Hocking, C. (2015) An occupational perspective of health (3 ^rd^ Ed.). Thorofare, NJ: SLACK Inc.

[ref155] Williams, N. H. and Law, R. J. (2018) Putting function first: redesigning the primary care management of long-term conditions. The British Journal of General Practice: The Journal of the Royal College of General Practitioners, 68, 388–389. 10.3399/bjgp18X698249 30049775 PMC6058632

[ref156] World Federation of Occupational Therapists (n.d.). About occupational therapy. https://wfot.org/about/about-occupational-therapy

