# Supplementary material for: Occupational therapy services in primary care: a scoping review
Source: Prim Health Care Res Dev. 2023 Jan 9;24:e7. doi: 10.1017/S1463423622000123 (PMC9884533; doi:10.1017/S1463423622000123)
Supplement: Supplementary file 1 [file S1463423622000123sup001.docx]

Appendix A: Full Electronic Search for OVID Medline database

1. occupational therapy/

2. "occupational therap*".tw,kf.

3. OT.ti.

4. or/1-3

5. physicians, primary care/

6. physicians, family/

7. general practitioners/

8. primary care nursing/

9. community health centers/

10. outpatients/

11. ambulatory care/

12. exp general practice/

13. exp primary health care/

14. ((general or primary or family) adj2 (physician* or doctor* or nurs* or practitioner*)).tw,kf.

15. ((ambulatory or general or primary or urgent or family) adj2 (care or healthcare or health care or medical care or patient care)).tw,kf.

16. outpatient*.tw,kf.

17. ((neighborhood or neighbourhood or community) adj3 (health or healthcare or center* or centre* or clinic?)).tw,kf.

18. or/5-17

19. 4 and 18
